# Identification of a Gene Prognostic Signature for Oral Squamous Cell Carcinoma by RNA Sequencing and Bioinformatics

**DOI:** 10.1155/2021/6657767

**Published:** 2021-04-01

**Authors:** Yang-Yang Zhang, Ming-Hui Mao, Zheng-Xue Han

**Affiliations:** Beijing Stomatological Hospital, Capital Medical University, Beijing 100050, China

## Abstract

**Objectives:**

Oral squamous cell carcinoma (OSCC) is the most common oral cancer and has a poor prognosis. We aimed to identify new biomarkers or potential therapeutic targets for OSCC.

**Materials and Methods:**

Four pairs of tumor and adjacent normal tissues were collected from OSCC patients, and differentially expressed genes (DEGs) were screened via high-throughput RNA sequencing (RNA-seq). Gene Ontology (GO) and Kyoto Encyclopedia of Genes and Genomes (KEGG) pathway enrichment analyses were used to analyze the DEGs. A protein-protein interaction (PPI) network was established with the Search Tool for the Retrieval of Interacting Genes/Proteins (STRING) database and Cytoscape, and two significant clusters were found. Candidate genes were screened by analyzing head and neck squamous cell carcinoma (HNSCC) data from The Cancer Genome Atlas (TCGA). A DEG-based risk model was established to predict the overall survival (OS) of OSCC patients via Kaplan-Meier analysis and the log-rank test. Furthermore, univariate Cox regression analysis was applied to assess associations between potential biomarkers and the overall survival rate.

**Results:**

Of 720 total DEGs, fifty-two DEGs in the two subclusters of the PPI network analysis were selected. A risk model was established, and five candidate genes (SPRR2E, ICOS, CTLA4, HTR1D, and CCR4) were identified as biomarkers of OS in OSCC patients.

**Conclusions:**

We successfully constructed a prognostic signature to predict prognosis and identified five candidate genes associated with the OS of OSCC patients that are potential tumor biomarkers and targets in OSCC.

## 1. Introduction

Oral squamous cell carcinoma (OSCC) is the most common type of head and neck malignant tumor, and >500,000 new cases of OSCC are diagnosed each year [1]. In addition, OSCC exhibits a high prevalence and morbidity, with 300,000 new cases and 145,000 deaths per year worldwide [2].

**Figure 1:**
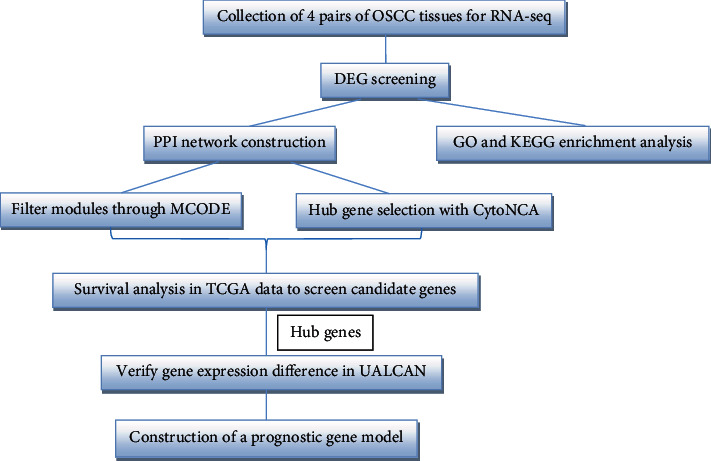
The workflow of this study.

The occurrence and development of OSCC are complex biological processes with the interaction and influence of multiple genes and factors [3]. Currently, there remains a lack of effective biomarkers or targets for OSCC therapy. Therefore, finding new tumor targets related to OSCC has become particularly important. High-throughput RNA sequencing (RNA-seq) has been widely used in cancer research. Moreover, bioinformatics is also playing an increasingly important role in this field, and the identification of differentially expressed genes (DEGs) has become a common analytical method for screening potential tumor markers. In this study, RNA-seq technology was used to compare tumorous and adjacent tissues. Through bioinformatics analysis, a series of DEGs related to OSCC were identified, laying the foundation for subsequent functional verification. The flow diagram of the present study is shown in Figure 1.

## 2. Materials and Methods

### 2.1. Collection of OSCC Specimens

This study was approved by the Ethics Committee of Beijing Stomatological Hospital of Capital Medical University. Written informed consent documents were obtained from all of the patients in our study.

A total of 4 pairs of OSCC tumor and adjacent tissues (at least 1.0 cm from the edge of the tumor) were collected at the Department of Oral and Maxillofacial-Head and Neck Oncology, Beijing Stomatological Hospital of Capital Medical University (patient details are shown in Table 1). Tissue specimens were fresh frozen immediately after surgery and stored at -80°C. All of the tumorous and normal adjacent tissues were confirmed as squamous cell carcinoma and normal tissues, respectively, by two pathologists from the Department of Diagnostic Pathology in our hospital. None of the patients had received preoperative chemotherapy, radiotherapy, or any other anticancer treatment prior to surgery.

### 2.2. High-Throughput RNA-seq

According to the manufacturer's protocol, total RNA was extracted from the four pairs of tissues using TRIzol (Invitrogen, Carlsbad, CA, USA). RNA integrity and concentration were assessed using the RNA Nano 6000 Assay. Sequencing libraries were generated using the NEBNext® Ultra™ RNA Library Prep Kit for Illumina® (#E7530L, NEB, USA) following the manufacturer's recommendations, and index codes were added to attribute sequences to each sample and then evaluated using the Agilent 2100 BioAnalyzer (Agilent Technologies, CA, USA). The DNA was purified, and sequencing was performed by the Illumina Cluster Station and Genome Analyzer (Illumina Inc., CA, USA) at the Beijing Genomics Institute, Shenzhen, according to the manufacturer's protocol.

### 2.3. Data Processing and DEG Screening

All of the DEGs were obtained through high-throughput RNA-seq. The expression value of genes between tumorous and adjacent normal tissues was compared with classical *t*-tests to identify DEGs. The false discovery rate (FDR) was used to adjust the *P* value and was analyzed by manipulating the FDR value [4]. The statistically significant DEGs were acquired by utilizing the “DEseq” package [5], and adjusted *P* value <0.05 and ∣log2 − fold change (FC) | ≥2 were set as the cut-off criteria [6].

### 2.4. Gene Enrichment Analysis

Gene Ontology (GO) and Kyoto Encyclopedia of Genes and Genomes (KEGG) pathway enrichment analyses of the DEGs [7] were performed via the Database for Annotation, Visualization and Integrated Discovery (DAVID) database (version 6.8, https://david. http://ncifcrf.gov/) to determine the functions of DEGs [8].

The false discovery rate (FDR) <0.05 was set as an inclusion criterion in both GO and KEGG enrichment analyses. Adjusted *P* value <0.05 was set as the cut-off criterion to identify enriched GO terms and KEGG pathways.

### 2.5. Protein-Protein Interaction (PPI) Network and Module Construction

The DEGs were enrolled in a PPI network using the STRING online software (http://www.string-db.org) [9], and all of the parameters were set as default values. Then, the PPI network was visualized using the Cytoscape software (version 3.7.1) [10]. Subsequently, module analysis of the PPI network was performed using the Molecular Complex Detection (MCODE) tool [11] in the Cytoscape software. Following the PPI analysis, the MCODE plugin was used to screen modules from the PPI network according to a k-core value of 2.

### 2.6. Survival Analysis to Screen the Candidate Genes

To validate and evaluate the prognostic value of the DEGs in the two highlighted module clusters, the human OSCC data were downloaded from The Cancer Genome Atlas (TCGA) database (https://cancergenome.nih.gov/). Furthermore, the screened genes were submitted to the UALCAN website (http://ualcan.path.uab.edu/index.html) [12] to analyze the gene expression data in the head and neck squamous cell carcinoma (HNSCC) dataset. According to gene median expression, the patients were divided into a high expression level group (with transcripts per million [TPM] values greater than the median) and a low-level group (with TPM values less than the median). The Kaplan-Meier method was used to determine which screening genes had significant prognostic value (*P* < 0.05).

### 2.7. Construction of a Prognostic Gene Model

The candidate genes were applied to build a predictive signature for survival. These prognostic genes from TCGA cohort were applied to a multivariate Cox regression model using Sangerbox tools, a free online platform for data analysis (http://www.sangerbox.com/tool), which contains the “Survival” and “ggplot2” packages for the R software. Multivariate analysis based on the linear combination of the expression levels of these hub genes was employed to calculate the prognostic risk score. The risk score for predicting overall survival (OS) was calculated using the following formula:

(1) $$\begin{array}{c} \text{Risk score}=\text{expression of gene}1\times \beta 1\text{gene}1\\ \;+\text{expression of gene}2\times \beta 2\text{gene}2\\ \;+\cdots \text{expression of gene}n\times \beta n\text{gene}n. \end{array}$$

^∗^The coefficient (*β*) was sourced from a multivariate Cox proportional hazards regression model for every gene.

Patients with available data from TCGA-OSCC cohort were then divided into high-risk and low-risk groups based on the prognostic risk score. The Kaplan-Meier method was performed, and the log-rank test was used to analyze the differences between the two groups. The predictive value and the sensitivity and specificity of the risk score were assessed by receiver operating characteristic (ROC) analysis. Furthermore, the prognostic value, including sensitivity and specificity of these five genes, was performed by ROC analysis.

### 2.8. Prognostic Value Assessment

We regrouped the patients according to the clinical parameters, such as age, gender, stage, TNM stage, neoplasm status, and risk level. Additionally, to evaluate the relationship between risk level and other clinical parameters, the chi-square test was performed. The SPSS Statistics software for Windows was used (version 22.0 IBM Corp., Armonk, NY) for data analysis. A *P* < 0.05 was considered a significant correlation. In clinical diagnosis and treatment, TNM stage is the major factors influencing prognosis. In this study, we aimed to assess the clinical application value of this risk score system. Thus, the clinical parameters with risk scores were analyzed in univariate Cox proportional hazards regression. Then, factors with a *P* < 0.05 were added into multivariate Cox proportional hazards regression. In this step, an index with a *P* < 0.05 can be considered an independent prognostic factor. The results of the Cox regression model analysis were more clearly exhibited by the forest plot R package provided by Sangerbox online platform.

## 3. Results

### 3.1. DEG Search and Analysis

**Figure 2:**
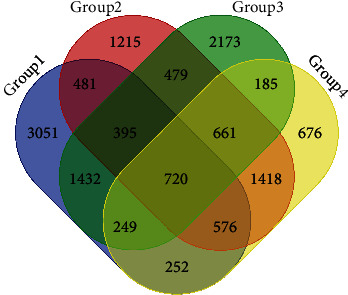
Identification of 720 commonly changed DEGs from the four groups. Different colored areas represent different groups. The overlapping areas indicate the commonly changed DEGs.

According to the sequencing data from the four pairs of OSCC and adjacent normal tissues, a total of 720 DEGs, including 451 upregulated and 269 downregulated genes, were screened out. As presented in Figure 2, different color areas represent different groups. The crossed areas indicate the commonly changed DEGs.

### 3.2. Gene Enrichment Analysis/Functional and Pathway Enrichment Analysis

**Figure 3:**
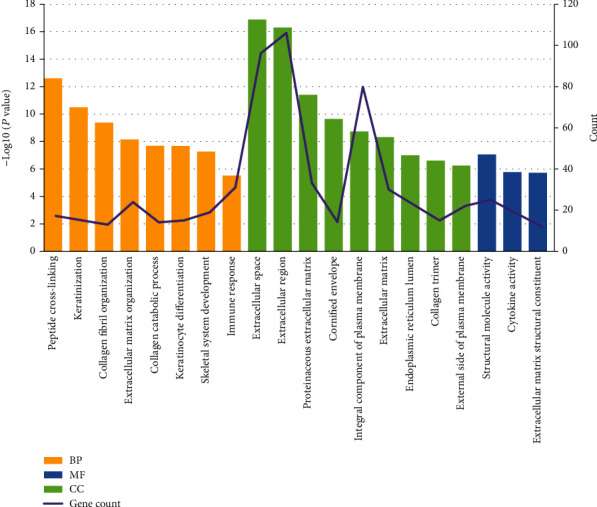
Significantly enriched GO terms of DEGs in OSCC versus adjacent tissues. GO analysis classified the DEGs into 3 groups (i.e., MF, BP, and CC). The DEGs were mostly correlated with the extracellular space, extracellular region, integral component of the plasma membrane, and peptide cross-linking terms.

**Figure 4:**
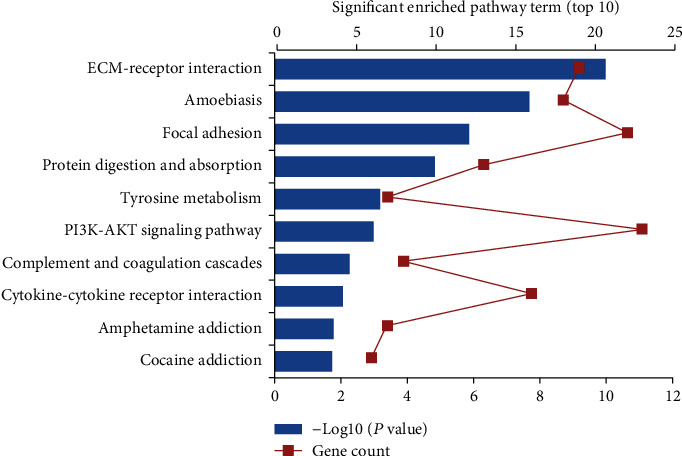
Significant enriched KEGG pathway terms of DEGs in OSCC. DEGs were enriched in ECM-receptor interaction, amoebiasis, and focal adhesion.

Based on the GO enrichment analysis, the commonly enriched categories were (i) extracellular space, (ii) extracellular region, (iii) peptide cross-linking, (iv) proteinaceous extracellular matrix, and (v) proliferation. The top 20 GO enrichment analysis results, including biological process (BP), cellular component (CC), and molecular function (MF) terms, are presented in Figure 3. In the BP analysis, the genes were significantly enriched in peptide cross-linking, keratinization, collagen fibril organization, extracellular matrix organization, collagen catabolic process, etc. In terms of cellular components, the genes were enriched in the extracellular space, extracellular region, proteinaceous extracellular matrix, cornified envelope, integral component of plasma membrane, extracellular matrix, etc. For the MF category, the genes were mainly enriched in the structural molecule activity, cytokine activity, and extracellular matrix structural constituent terms. According to KEGG pathway analysis, our results indicated that these genes were significantly enriched in extracellular matrix- (ECM-) receptor interaction, amoebiasis, focal adhesion, protein digestion and absorption, tyrosine metabolism, etc. (Figure 4).

### 3.3. Identification of Module Clusters via PPI Network Analysis

**Figure 5:**
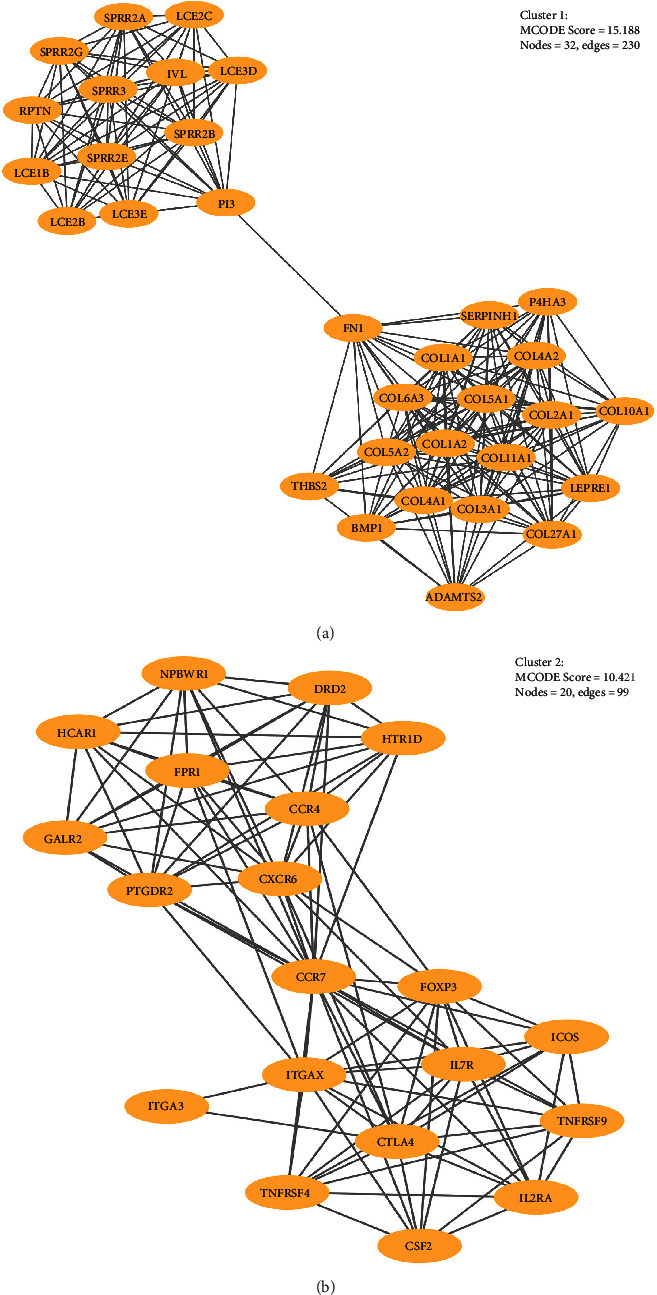
(a) The hub gene in cluster 1; (b) the hub gene in cluster 2.

A total of 444 nodes and 1555 interactions were selected; the 444 nodes included 251 upregulated genes, 186 downregulated genes, and 13 genes without significant log2FC values, which were filtered into the DEG PPI network complex (Figure S1). According to module analysis, two highlighted module clusters were discovered: cluster 1 consisted of 32 nodes (COL4A1, COL1A2, COL1A1, COL5A2, COL3A1, COL11A1, COL5A1, COL6A3, COL2A1, COL4A2, COL27A1, LEPRE1, SERPINH1, FN1, COL10A1, P4HA3, ADAMTS2, PI3, BMP1, IVL, SPRR3, SPRR2A, SPRR2E, LCE2C, LCE2B, LCE3E, SPRR2B, LCE1B, LCE3D, SPRR2G, RPTN, and THBS2) and 230 edges, which were mainly associated with peptide cross-linking, cornified envelope, collagen catabolic process, etc. (Figure 5, Table S1). Cluster 2 consisted of 20 nodes (CCR7, CXCR6, ITGAX, CTLA4, CCR4, FOXP3, IL7R, PTGDR2, FPR1, HCAR1, IL2RA, TNFRSF9, HTR1D, DRD2, NPBWR1, GALR2, TNFRSF4, CSF2, ICOS, and ITGA3) and 99 edges, which were mainly associated with the external side of the plasma membrane, immune response, plasma membrane, etc. (Figure 5, Table S2). The density of the PPI network was confirmed with the high degree of nodes, suggesting competition in OSCC.

### 3.4. Survival Analysis

**Figure 6:**
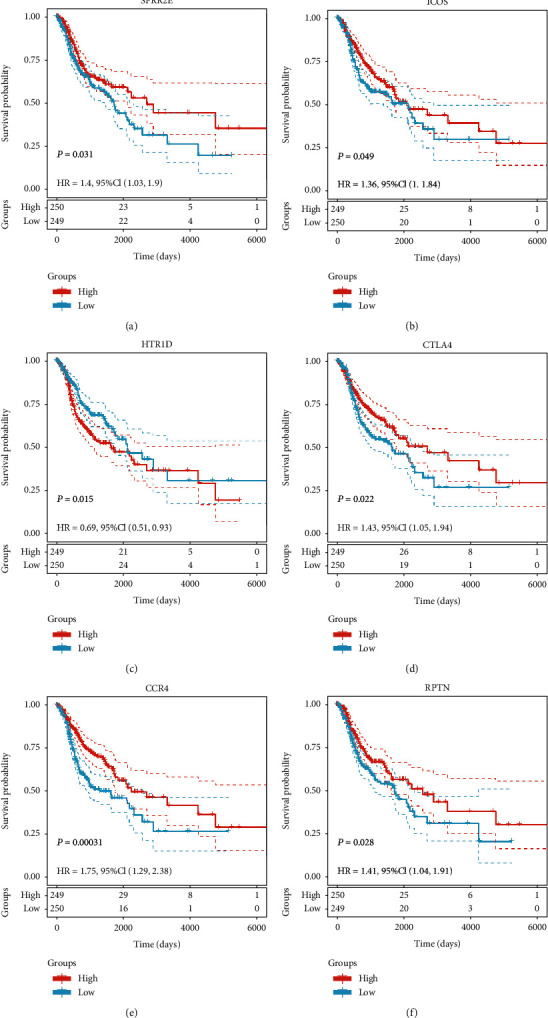
OS associated with the six key genes in OSCC based on the Kaplan-Meier plotter. (a–f) Red plots present high expression of each individuals; blue plots present median/low expression of each individuals. The dashed line represents 95% confidence interval.

**Figure 7:**
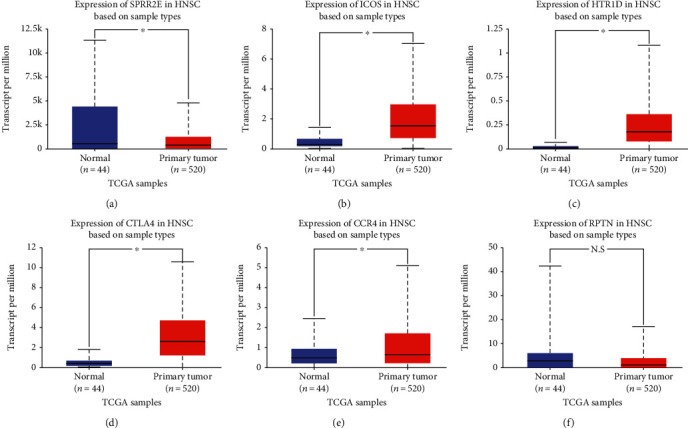
The transcription level of the six key genes in OSCC according to UALCAN. (a–f) Expression levels of SPRR2E, ICOS, HTR1D, CTLA4, and CCR4 were significant differentially expressed between tumorous and adjacent normal tissues in HNSCC (black star represents *P* value <0.05; N.S represents no significance).

A total of 499 patients with available data from TCGA-OSCC dataset were included in the individual survival analysis. The OS of patients with OSCC based on the high and low expression of the hub DEGs was analyzed by the log-rank test. The survival analysis results revealed that high expression of SPRR2E, ICOS, CTLA4, HTR1D, RPTN, and CCR4 was associated with poor OS in patients with OSCC (*P* < 0.05; Figures 6(a)–6(f)). Then, we used UALCAN to analyze the expression levels of the six candidate genes of OSCC (*P* < 0.05; Figures 7(a)–7(f)). Finally, SPRR2E, ICOS, CTLA4, HTR1D, and CCR4 were selected as key candidate genes (Table 2).

### 3.5. Construction of the Prognostic Signature Based on Candidate Genes

We identified 5 prognosis-related genes, including SPRR2E, ICOS, CTLA4, HTR1D, and CCR4, and performed a multivariate Cox proportional hazards regression analysis on these candidate genes to determine whether the signatures exhibited significant prognostic value. The risk score for predicting OS was calculated as follows:

(2) $$\begin{array}{c} \text{Risk score}=\text{expression of SPRR}2\text{E}\times \left(‐0.000076\right)\\ \;+\text{expression of CCR}4\times \left(‐0.02217\right)\\ \;+\text{expression of ICOS}\times \left(‐0.02666\right)\\ \;+\text{expression of CTLA}4\times \left(0.010051\right)\\ \;+\text{expression of HTR}1\text{D}\times \left(0.035233\right). \end{array}$$

**Figure 8:**
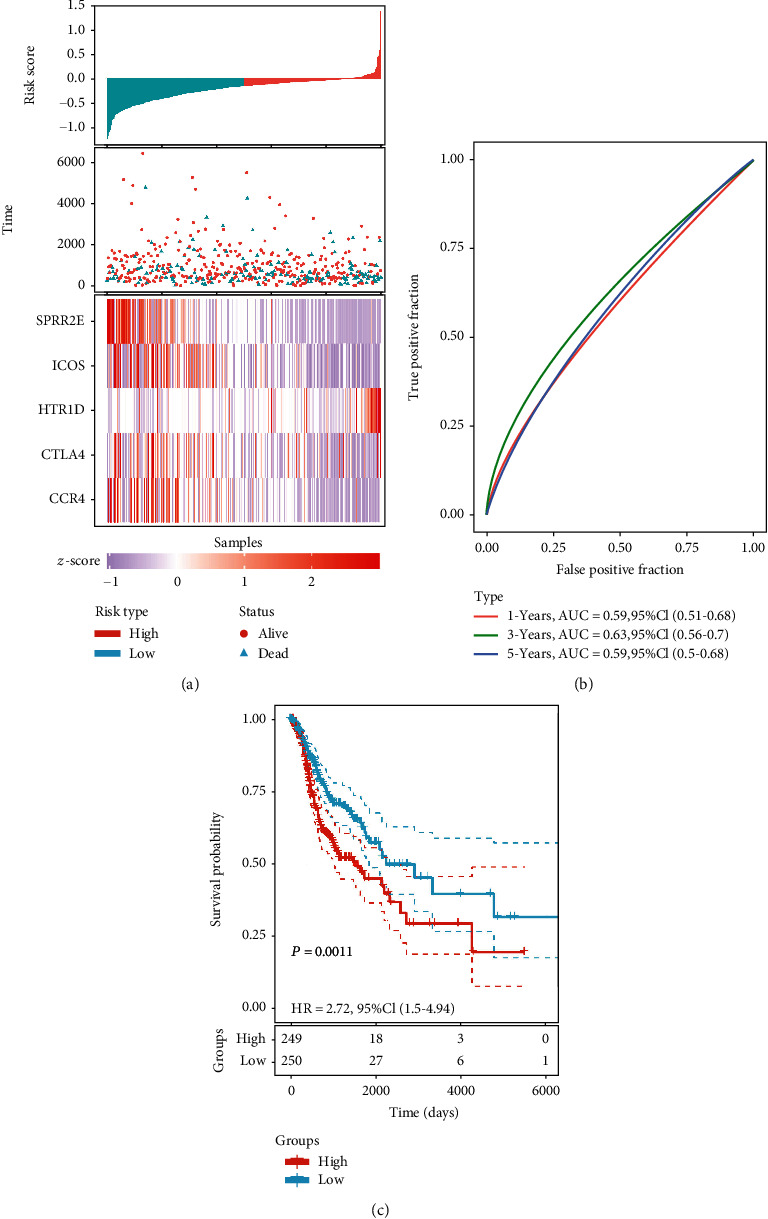
Identification of prognosis-related genes in 499 OSCC patients. (a) The risk score, patient survival time, patient survival status, and expression of the genes in the five-gene signature. (b) ROC curves and AUC values indicating the ability of the five-gene signature to classify patients as high risk or low risk. (c) Kaplan-Meier survival analysis of patients according to the risk score from the five-gene signature. In total, 249 patients were assigned to the high-risk group, and 250 patients were assigned to the low-risk group. The dashed line represents 95% confidence interval.

**Figure 9:**
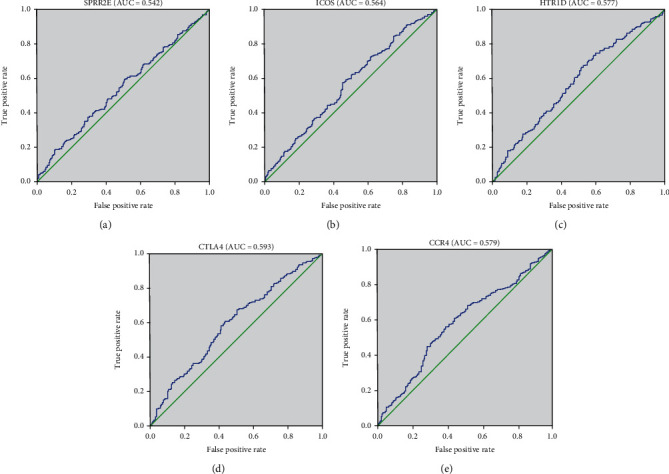
Receiver operating characteristic curve of 5 genes. ROC was performed for SPRR2E, ICOS, HTR1D, CTLA4, and CCR4 for the prognostic value in HNSCC.

According to the risk score, the samples were divided into a high-risk group and a low-risk group. High expression levels of CTLA4 and HTR1D were associated with high risk and thus were risk factors, while low expression levels of SPRR2E, ICOS, and CCR4 were protective factors (Figure 8(a)). ROC analysis of the prognostic classification of risk score was further performed, and the average 1-, 3-, and 5-year area under the curve (AUC) values for the five-gene signature was 0.59, 0.63, and 0.59, respectively (Figure 8(b)). A total of 249 samples were sorted into the high-risk group, and 250 were sorted into the low-risk group. The prognoses of the high-risk and low-risk groups significantly differed according to the log-rank test (*P* = 0.0011 and HR = 2.72 [1.50-4.94]) (Figure 8(c)). The prognostic value, including sensitivity and specificity of SPRR2E, ICOS, HTR1D, CTLA4, and CCR4, was shown in Figure 9.

### 3.6. Prognostic Value Assessment

As shown in Table 3, clinical parameters, consisting of age, gender, stage, TNM stage, neoplasm status, vital status and risk level, were divided into groups. In the chi-square test, risk level has a significant correlation with gender (*P* = 0.001), neoplasm status (*P* = 0.029), and vital status (*P* = 0.032) (Table 4).

**Figure 10:**
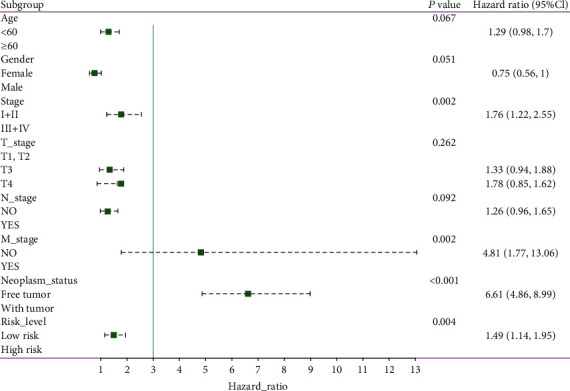
Forest map of univariate logistic regression analysis. The line shows the 95% CI, and the location of the green square on the line represents the odds ratio.

**Figure 11:**
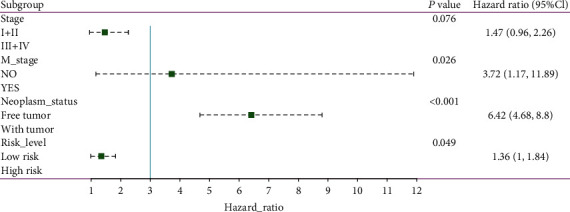
Forest map of multivariate logistic regression analysis. The line shows the 95% CI, and the location of the green square on the line represents the odds ratio.

To compare the prognostic power of the risk score system with clinical parameters, univariate Cox proportional hazards regression analysis was performed. We found that tumor stage, M stage, neoplasm status, and risk level are indicators of poor outcomes (Figure 10). Additionally, these four indices were entered into multivariate Cox proportional hazards regression analysis. The M stage, neoplasm status, and risk level had a *P* <0.05, indicating that they can be utilized as independent factors in evaluating clinical outcomes (Figure 11).

## 4. Discussion

OSCC is a type of malignant tumor with high morbidity and mortality. In addition, its well-known characteristics of easy recurrence and distant metastasis contribute to its poor prognosis. In addition to traditional surgical treatment and/or radiotherapy and chemotherapy, gene therapy has received extensive attention and research. However, the key biomarkers or target genes for OSCC therapy are currently insufficient, so it is necessary to identify novel biomarkers and ways to treat OSCC. In recent years, bioinformatics has played an increasingly important role in cancer research and has become a common method for screening important DEGs in tumors. For example, Zou et al. [13] obtained 3 OSCC-related DEGs by bioinformatics analysis of 4 public datasets, including 244 OSCC tumors and 95 normal controls. Wang et al. [14] identified six candidates genes closely related to the survival rate of patients with oral cancer through comprehensive bioinformatics and further confirmed DEGs between OSCC tumors and normal controls by q-PCR. Kisoda et al. [15] used public RNA sequence data of 519 primary HNSCC cases obtained from TCGA database to examine the prognostic value of p-EMT-related genes and revealed that six DEGs could be used as prognostic markers for HNSCC. However, most studies have focused only on genes in public databases and rarely combine clinical samples for comprehensive screening or established prognostic survival models. In this study, we did not conduct isolated bioinformatics analysis but first performed RNA-seq on the tumor samples of OSCC patients in our hospital and then combined these data with the available HNSCC data from large TCGA datasets to identify biomarkers in OSCC, providing more reliable and accurate results.

In the present study, 720 DEGs in OSCC versus adjacent normal tissues were obtained. GO analysis showed that the DEGs were mainly related to the extracellular space, which is the space outside of the cell membrane that is a part of multicellular organisms; this term typically indicates the involvement of a secreted proteins that remain associated with the cell, e.g., as part of the ECM (extracellular matrix). The KEGG pathway enrichment analysis indicated that DEGs were mainly enriched in ECM-receptor interactions. The ECM, a highly dynamic structure, can continuously undergo remodeling, including ECM component deposition, degradation, or other modifications [16]. Consistent with the GO analysis results, the KEGG analysis results showed that ECM-receptor interaction was one of the most significantly enriched categories. The enrichment of the ECM-receptor interaction signaling pathway might be related to the expression of integrin genes (ITGs) [17], which play important roles in the connection between cells and extracellular environments and are important cell-surface receptors, and they have been demonstrated as playing critical roles in the progression of OSCC [18]. For instance, the integrin-*α*5 (ITGA5) gene has been reported to promote the progression of OSCC by activating the PI3K/AKT signaling pathway [19].

A PPI network was constructed to obtain two subclusters. Six genes (SPRR2E, ICOS, CTLA4, HTR1D, RPTN, and CCR4) that were closely related to the OS of patients were identified by analyzing the survival data of patients with HNSCC from TCGA. Expression verification of these central genes was performed by UALCAN according to TCGA database. Through this analysis process, five candidate genes (SPRR2E, CCR4, CTLA4, HTR1D, and ICOS) were screened out. The prognostic signature based on the five candidate genes can predict the prognosis of OSCC; thus, these genes might be potential biomarkers for OSCC. Notably, some previous studies have revealed their important role in cancer.

SPRR2E, one of the seven SPRR2 genes (A-G), encodes a member of a family of small proline-rich proteins clustered in the epidermal differentiation complex on chromosome 1q21. The encoded protein, along with other family members, is a component of the cornified cell envelope that forms beneath the plasma membrane in terminally differentiated stratified squamous epithelia. This envelope serves as a barrier against extracellular and environmental factors. It has been reported that the SPRR protein can provide a barrier for the epithelium and can adapt to various external injuries [20]. Currently, there have been few studies of SPRR2E, and further research is required.

ICOS plays an important role in cell-cell signaling, immune responses, and cell proliferation regulation. Previous studies have reported that ICOS is widely expressed in cutaneous T cell lymphoma and can target and potently kill malignant cells [21]. Notably, ICOS encoded by this gene belongs to the CD28 and CTLA4 cell-surface receptor family. CTLA4 is also one candidate gene screened in this study. It is a member of the immunoglobulin superfamily and encodes a protein that transmits an inhibitory signal to T cells. A recent study showed that CTLA4, as a tumor suppressor gene, has a vital function in suppressing the immune responses of activated T lymphocytes [22]. The recurrence-free survival and metastasis-free survival rates were decreased in patients with large numbers in the parenchyma of the invasive front compared to those with low number of CTLA4^+^ cells in this area [23]. It has also been reported that ICOS and CTLA4 lead to disturbances of the immune response and thereby indicate an increased risk of cancer [24].

HTR1D (5-HT1D), an indispensable member of the serotonergic system, has been demonstrated to play an important role in the regulation of the proliferative and invasive phenotype of pancreatic cancer (PaCa), and downregulation of 5-HT1D receptors inhibits proliferation and invasion of human pancreatic cancer cells [25]. Some studies have shown that 5-HT1D promotes hepatocellular carcinoma (HCC) proliferation [26]; in addition, downregulation of 5-HT1D affects HCC progression [27]. Upregulation of HTR1D in HCC tissues predicts poor overall survival and high recurrence probability in HCC patients [28]. Unfortunately, no studies have discussed the regulatory role of HTR1D in OSCC, so further research is needed.

CCR4 is a gene that is well-known, as a CCX-type receptor expressed on various immune cells, especially on T-helper type 2 cells [29], and several investigations have indicated that CCR4 plays a key role in the metastasis of OSCC [30–32]. A recent study reported that CCR4 in oral tongue tissues was positively correlated with poor prognosis of patients with pN0 and emphasized its potential as a prognostic biomarker and therapeutic target in oral tongue cancer [33].

Although we used RNA-seq and bioinformatics technology to analyze clinical samples and identified potential candidate genes that could affect tumor prognosis, this study still has some limitations: (a) the patient sample size for RNA-seq analysis in this study was small; (b) in the survival analysis of this study, the tumor pathological type and grade were not used to verify the survival analysis, but this fact might be due to no detailed relevant information in the database; (c) furthermore, in this survival analysis, we did not consider the impact of the patient's economic status, health insurance coverage, treatment method, etc., all of which significantly influence clinical outcomes; and (d) the results of the bioinformatics analysis must to be confirmed by experimental verification, which will be the focus of our next research study.

## 5. Conclusion

Generally, TNM stage and neoplasm status are used in identifying high-risk patients, but these factors are difficult to utilize in clinical diagnosis and treatment without a quantitative index. This study reports the successful construction of a prognostic signature to predict the prognosis of OSCC patients, and five candidate genes were revealed to be associated with OS, suggesting that they might be potential tumor biomarkers or targets for OSCC. In addition, further studies, such as those with a larger sample size and longer follow-up of clinical cases, are needed to reveal and verify the pathogenesis and prognosis of OSCC.

## Figures and Tables

**Table 1:** The correlation of OSCC clinical-pathological variables.

Variable	No. of cases/mean ± SD
Age	58.37 ± 12.21
Male	2
Location	
Ventral tongue	1
Tongue border	2
Tongue base	1
Tumor morphology	
Ulcer type	2
Invasive type	1
Exogenous type	1
Stage	
I+II	1
III+IV	3
T stage	
Tis, T1, T2	3
T3	1
T4	0
N stage	
N0	1
N1+N2	3
M stage	
M0	4
M1	0

**Table 2:** The candidate DEGs screened out by survival analysis were significantly correlated with overall survival in HNSCC patients.

Gene ID	Description	Ensemble ID	*P* value	HR (95% CI)
SPRR2E	Small proline-rich protein 2E	ENSG00000203785	0.031	1.4 (1.03-1.90)
ICOS	Inducible T cell costimulator	ENSG00000163600	0.049	1.36 (1.00-1.84)
HTR1D	5-Hydroxytryptamine receptor 1D	ENSG00000179546	0.015	0.69 (0.51-.093)
CTLA4	Cytotoxic T lymphocyte-associated protein 4	ENSG00000163599	0.022	1.43 (1.05-1.94)
CCR4	C-C motif chemokine receptor 4	ENSG00000183813	0.00031	1.75 (1.29-2.38)

**Table 3:** The clinical parameters of HNSCC patients.

Subgroup	Frequency	Percentage	Valid percentage
Age			
<60	220	44.1	44.1
≥60	279	55.9	55.9
Gender			
Male	366	73.3	73.3
Female	133	26.7	26.7
Stage			
I+II	105	21.0	21.0
III+IV	394	79.0	79.0
T stage			
Tis, T1, T2	176	35.3	36.1
T3	132	26.5	27.0
T4	180	36.1	36.9
N stage			
N0	240	48.1	50.0
N1+N2	240	48.1	50.0
M stage			
M0	474	95.0	99.0
M1	5	1.0	1.0
Neoplasm status			
Tumor free	314	62.9	69.6
With tumor	137	27.5	30.4
Vital status			
Alive	282	56.5	56.5
Dead	217	43.5	43.5
Risk level			
High	249	49.9	49.9
Low	250	50.1	50.1

**Table 4:** Relationship between risk level and clinical parameters; ^∗^represents significant *P* value.

Subgroup	High risk	Low risk	Total	*P*
Age				0.447
<60	114 (22.85%)	106 (21.24%)	220	
≥60	135 (27.05%)	144 (28.86%)	279	
Gender				0.001^∗^
Male	199 (39.88%)	167 (33.47%)	366	
Female	50 (10.02%)	83 (16.63%)	133	
Stage				0.073
I+II	47 (9.42%)	58 (11.62%)	105	
III+IV	202 (40.48%)	192 (38.48%)	394	
T stage				0.454
Tis, T1, T2	87 (17.83%)	89 (18.24%)	176	
T3	61 (12.50%)	71 (14.55%)	132	
T4	96 (19.67%)	84 (17.21%)	180	
N stage				0.784
N0	122 (25.42%)	118 (24.58%)	240	
N1+N2	119 (24.79%)	121 (25.21%)	240	
M stage				0.677
M0	234 (48.85%)	240 (50.10%)	474	
M1	2 (0.42%)	3 (0.63%)	5	
Neoplasm status				0.029^∗^
Tumor free	146 (32.37%)	168 (37.25%)	314	
With tumor	79 (17.52%)	58 (12.86%)	137	
Vital status				0.032^∗^
Alive	130 (26.05%)	152 (30.46%)	282	
Dead	119 (23.85%)	98 (19.64%)	217	

## Data Availability

(1) The datasets used and/or analyzed during the current study are available from the public database on reasonable request. (2) The detailed information regarding the RNA-seq data had been uploaded to NCBI (BioProject ID: PRJNA701130). The reviewer link is https://dataview.ncbi.nlm.nih.gov/object/PRJNA701130?reviewer=sbjvsipsvttcuvbk7jp95fibqt.

## Supplementary Materials

Supplementary Materials Figure S1: the protein-protein interaction (PPI) of DEGs. Darker node corresponds to higher 453 degrees of DEGs. The node sizes are from small to large according to the betweenness 454 centrality (from low to high). Table S1: GO and KEGG pathway analyses of DEGs in module 1. The top five GO items in BP, CC, and MF and the top five significant enriched pathway terms. Table S2: GO and KEGG pathway analyses of DEGs in module 2. The top five GO items in BP, CC, and MF and the top five significant enriched pathway terms.
