# Supplementary material for: Identification of a Gene Prognostic Signature for Oral Squamous Cell Carcinoma by RNA Sequencing and Bioinformatics
Source: Biomed Res Int. 2021 Apr 1;2021:6657767. doi: 10.1155/2021/6657767 (PMC8032525; doi:10.1155/2021/6657767)
Supplement: Supplementary Materials — Figure S1: the protein-protein interaction (PPI) of DEGs. Darker node corresponds to higher 453 degrees of DEGs. The node sizes are from small to large according to the betweenness 454 centrality (from low to high). Table S1: GO and KEGG pathway analyses of DEGs in module 1. The top five GO items in BP, CC, and MF and the top five significant enriched pathway terms. Table S2: GO and KEGG pathway analyses of DEGs in module 2. The top five GO items in BP, CC, and MF and the top five significant enriched pathway terms. [file 6657767.f1.zip › TableS1.docx]

**Table S1** GO and KEGG pathways analysis of module 1

|  | **GO.ID** | **Term** | **Count** | **P-value** |
| --- | --- | --- | --- | --- |
| BP | GO:0018149 | peptide cross-linking | 13 | 5.80E-24 |
|  | GO:0030574 | collagen catabolic process | 12 | 3.25E-20 |
|  | GO:0031424 | keratinization | 11 | 2.56E-19 |
|  | GO:0030216 | keratinocyte differentiation | 11 | 3.62E-17 |
|  | GO:0030198 | extracellular matrix organization | 13 | 1.96E-16 |
| CC | GO:0001533 | cornified envelope | 12 | 3.82E-22 |
|  | GO:0005788 | endoplasmic reticulum lumen | 14 | 1.32E-18 |
|  | GO:0005581 | collagen trimer | 11 | 1.78E-16 |
|  | GO:0005578 | proteinaceous extracellular matrix | 11 | 9.28E-12 |
|  | GO:0031012 | extracellular matrix | 11 | 2.48E-11 |
| MF | GO:0005201 | extracellular matrix structural constituent | 10 | 1.86E-15 |
|  | GO:0048407 | platelet-derived growth factor binding | 6 | 5.72E-12 |
|  | GO:0005198 | structural molecule activity | 11 | 8.71E-12 |
|  | GO:0046332 | SMAD binding | 3 | 0.002635 |
|  | GO:0030674 | protein binding, bridging | 3 | 0.008224 |
|  | **Pathways** | **Description** |  |  |
| KEGG Pathway | hsa04512 | ECM-receptor interaction | 13 | 6.80E-22 |
|  | hsa04974 | Protein digestion and absorption | 12 | 2.78E-19 |
|  | hsa04510 | Focal adhesion | 13 | 3.26E-17 |
|  | hsa05146 | Amoebiasis | 11 | 4.64E-16 |
|  | hsa04151 | PI3K-Akt signaling pathway | 13 | 1.75E-14 |
