# Supplementary material for: Identification of a Gene Prognostic Signature for Oral Squamous Cell Carcinoma by RNA Sequencing and Bioinformatics
Source: Biomed Res Int. 2021 Apr 1;2021:6657767. doi: 10.1155/2021/6657767 (PMC8032525; doi:10.1155/2021/6657767)
Supplement: Supplementary Materials — Figure S1: the protein-protein interaction (PPI) of DEGs. Darker node corresponds to higher 453 degrees of DEGs. The node sizes are from small to large according to the betweenness 454 centrality (from low to high). Table S1: GO and KEGG pathway analyses of DEGs in module 1. The top five GO items in BP, CC, and MF and the top five significant enriched pathway terms. Table S2: GO and KEGG pathway analyses of DEGs in module 2. The top five GO items in BP, CC, and MF and the top five significant enriched pathway terms. [file 6657767.f1.zip › TableS2.docx]

**Table S2** GO and KEGG pathways analysis of module 2

|  | **GO.ID** | **Term** | **Count** | **P-value** |
| --- | --- | --- | --- | --- |
| BP | GO:0006955 | immune response | 10 | 2.66E-10 |
|  | GO:0006954 | inflammatory response | 7 | 2.69E-06 |
|  | GO:0006935 | chemotaxis | 5 | 9.45E-06 |
|  | GO:0050777 | negative regulation of immune response | 3 | 7.95E-05 |
|  | GO:0048872 | homeostasis of number of cells | 3 | 9.35E-05 |
| CC | GO:0009897 | external side of plasma membrane | 9 | 2.07E-11 |
|  | GO:0005886 | plasma membrane | 17 | 2.17E-08 |
|  | GO:0005887 | integral component of plasma membrane | 12 | 2.51E-08 |
|  | GO:0005622 | intracellular | 6 | 0.0101137 |
|  | GO:0009986 | cell surface | 4 | 0.01778 |
| MF | GO:0042923 | neuropeptide binding | 3 | 2.02E-04 |
|  | GO:0016493 | C-C chemokine receptor activity | 2 | 0.012725 |
|  | GO:0005031 | tumor necrosis factor-activated receptor activity | 2 | 0.025297 |
|  | GO:0004930 | G-protein coupled receptor activity | 4 | 0.037739 |
|  | GO:0008144 | drug binding | 2 | 0.078048 |
|  | **Pathways** | **Description** |  |  |
| KEGG Pathway | hsa04060 | Cytokine-cytokine receptor interaction | 8 | 1.44E-06 |
|  | hsa04640 | Hematopoietic cell lineage | 4 | 0.00139 |
|  | hsa04080 | Neuroactive ligand-receptor interaction | 5 | 0.005033 |
|  | hsa04660 | T cell receptor signaling pathway | 3 | 0.027514 |
|  | hsa04630 | Jak-STAT signaling pathway | 3 | 0.054158 |
